# Two-photon-like microscopy with orders-of-magnitude lower illumination intensity via two-step fluorescence

**DOI:** 10.1038/ncomms9184

**Published:** 2015-09-03

**Authors:** Maria Ingaramo, Andrew G. York, Eric J. Andrade, Kristin Rainey, George H. Patterson

**Affiliations:** 1 National Institute of Biomedical Imaging and Bioengineering, National Institutes of Health, Bethesda, Maryland 20892, USA.

## Abstract

We describe two-step fluorescence microscopy, a new approach to non-linear imaging based on positive reversible photoswitchable fluorescent probes. The protein Padron approximates ideal two-step fluorescent behaviour: it equilibrates to an inactive state, converts to an active state under blue light, and blue light also excites this active state to fluoresce. Both activation and excitation are linear processes, but the total fluorescent signal is quadratic, proportional to the square of the illumination dose. Here, we use Padron’s quadratic non-linearity to demonstrate the principle of two-step microscopy, similar in principle to two-photon microscopy but with orders-of-magnitude better cross-section. As with two-photon, quadratic non-linearity from two-step fluorescence improves resolution and reduces unwanted out-of-focus excitation, and is compatible with structured illumination microscopy. We also show two-step and two-photon imaging can be combined to give quartic non-linearity, further improving imaging in challenging samples. With further improvements, two-step fluorophores could replace conventional fluorophores for many imaging applications.

## Abstract (web summary)

Two-photon fluorescence gives a quadratic response, which improves imaging in thick samples but requires extremely intense illumination. Here, the authors describe two-step fluorescent imaging, a much lower intensity approach to quadratic excitation, via reversible photoswitchable fluorophores.

## Graphical abstract

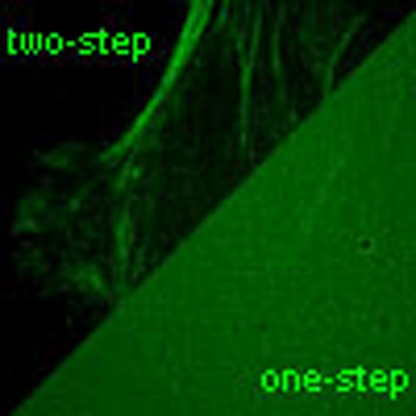

Modern fluorescence microscopy is invaluable to biologists, visualizing live cellular processes with high resolution, high speed and molecular specificity. Advances in fluorescent probes, especially genetically expressed probes like green fluorescent protein, are critical for advancing fluorescence microscopy^1^,^2^. Brighter, more biocompatible probes allow biologists to see more clearly for longer durations, while probes with unusual photophysical behaviour enable a wide variety of new imaging techniques.

Typical fluorescent probes respond linearly to excitation, meaning fluorescent signal is proportional to illumination dose. Probe response can be non-linear in certain conditions, due to effects like fluorescence saturation, stimulated emission, stochastic photoswitching or saturated photoswitching. Many microscopy techniques exploit these non-linearities to greatly improve image resolution^3^,^4^,^5^,^6^,^7^,^8^,^9^,^10^. These ‘superresolution’ techniques typically require specialized hardware, software and/or sample preparation, and work best for highly transparent, photostable samples.

Alternatively, non-linearity can enable robust normal-resolution imaging in challenging samples. Quadratic non-linearity (fluorescent signal proportional to the square of illumination intensity) is particularly useful for thicker, less transparent samples like animal tissue^11^,^12^. A quadratic response effectively vanishes for weak illumination, which slightly improves resolution by shrinking the excitation volume, but strongly suppresses excitation due to scattered or out-of-focus illumination. Reduced out-of-focus photobleaching allows many more *z* planes per image volume, and if scattering is low, fluorescent emission can be imaged to a pinhole to further reduce out-of-focus background in thick samples. If scattering is high, the excitation region is less deformed by illumination scattering, and emission scattering can be ignored because the excitation alone produces a sectioned image.

Modifying a confocal microscope to use quadratic excitation is straightforward, via two-photon fluorescence. Doubling the illumination wavelength prevents linear excitation, and two-photon excitation (a quadratic process) dominates. This approach has positive and negative side effects: longer wavelengths scatter less in many biological samples^13^ but cannot focus as tightly, and most importantly, cross-sections for two-photon excitation are extremely low while photobleaching is high. Switching from one-photon to two-photon excitation requires more expensive, less-reliable-pulsed lasers with orders-of-magnitude higher peak (∼10^8^-fold) and average (∼10^3^-fold) intensities to achieve similar excitation rates^14^, and has been observed^15^ to cause substantially worse photobleaching per excitation event.

Here, we propose and demonstrate an alternative quadratic excitation method, which we call two-step fluorescence. An ideal two-step fluorophore would give all the benefits of quadratic excitation on an unmodified confocal microscope, without the tradeoffs of two-photon excitation. To our knowledge, no ideal two-step fluorophore exists. However, we show that under appropriate conditions, the positive reversible photoswitchable fluorescent protein ‘Padron’^16^ closely approximates a two-step fluorophore. We use Padron to demonstrate how two-step imaging can improve resolution and dramatically improve sectioning in biological samples. We also show that two-step fluorescence combined with two-photon excitation gives a quartic signal (proportional to intensity to the fourth power), further improving sectioning in challenging samples where imaging is otherwise nearly impossible.

## Results

### Ideal two-step fluorescence would give quadratic non-linearity

**Figure 1:**
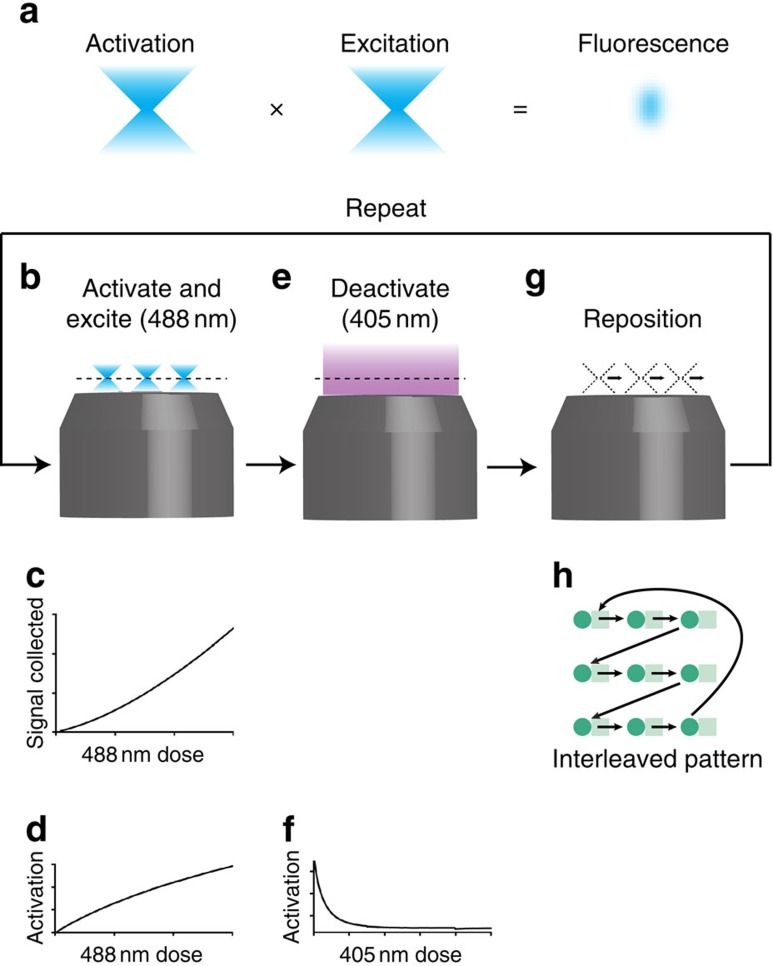
Two-step fluorescence principle and two-step imaging using Padron. (**a**) Activation and excitation probabilities are proportional to illumination intensity for an ideal two-step fluorophore. Fluorescence is confined to a small region, the product of the activation and excitation regions. (**b**) We activate and excite Padron with point-focused 488 nm light, and image the fluorescence onto a camera. (**c**) The signal collected is approximately quadratic in the 488 nm dose, because (**d**) the degree of activation is approximately linear in the 488 nm dose for our typical dosage. (**e**) Next we deactivate Padron with an unfocused beam of 405 nm light, to mimic the spontaneous deactivation of an ideal two-step fluorophore. (**f**) The activated fraction decays exponentially with increasing 405 nm dose. (**g**) Finally we reposition the illumination pattern, and repeat the process. The scan pattern is coarse to avoid exciting previously activated Padron, and (**h**) we interleave multiple coarse scans to achieve a fine scan. Note that panels (**c**,**d**,**f**) are conceptual illustrations; for quantitative information, see Supplementary Fig. 4 and ref. 16.

An ideal two-step fluorophore would have two states, active and inactive, and would rapidly equilibrate to the inactive state. Illumination activates the fluorophore, and also excites the active state, causing fluorescence proportional to the degree of activation multiplied by the degree of excitation, as illustrated in Fig. 1a and Supplementary Fig. 1. If both activation and excitation probabilities are proportional to illumination intensity, and neither activation nor excitation approaches 100%, then the signal is quadratic, proportional to the illumination squared.

### Padron can imitate an ideal two-step fluorophore

Padron has many of the properties of an ideal two-step fluorophore. Blue light switches Padron to an active state, which fluoresces strongly under blue excitation; Padron equilibrates to an inactive state, and allows hundreds of switching cycles. Unfortunately, equilibration to the inactive state takes hours, and inactive Padron also fluoresces (weakly) under blue excitation. Fortunately, violet light efficiently deactivates Padron, and Padron’s contrast ratio (active fluorescence divided by inactive fluorescence^17^) can exceed 100. Using blue light for simultaneous activation/excitation and violet light to control active-state lifetime makes high-contrast Padron act as a nearly ideal two-step fluorophore, allowing us to demonstrate the benefits of two-step imaging.

### Imaging procedure

Figs 1b–h illustrates our two-step fluorescent imaging procedure:

First, illuminate the sample with point-focused blue light (Fig. 1b). This activates and excites Padron causing fluorescent emission, which we image onto a camera. Like conventional microscopy, we ensure our illumination does not saturate excitation, and we also ensure our illumination does not saturate activation (Fig. 1d). Thus, activation and excitation are both nearly linear and fluorescence is nearly quadratic (Fig. 1c). We use many illumination spots in parallel to speed acquisition.

Next, deactivate the sample with widefield violet light (Fig. 1e). Two-step imaging with Padron requires violet light to control the active state’s lifetime (Fig. 1f). Unfortunately, violet light also excites fluorescence in our samples, so we deactivate between camera exposures. This step would be unnecessary for an ideal two-step fluorophore, which would spontaneously deactivate more rapidly. Also, an ideal two-step fluorophore’s deactivation rate would be independent of its position; the violet illumination is widefield to mimic this ideal behaviour.

Then, reposition the point-focused illumination (Fig. 1g). The illumination pattern moves to the next position of a coarse raster scan. Only the blue light is scanned; the violet light has nearly uniform intensity, to give Padron an activated lifetime independent of position like an ideal two-step fluorophore.

Finally, repeat (Fig. 1h). We interleave multiple coarse scans to produce a fine scan; this gives Padron more time to deactivate between successive illuminations. Overlap between illumination and residual activation could degrade resolution and sectioning.

Padron also allows linear (one-step) fluorescence imaging. The procedure is similar, except we preactivate Padron using widefield blue light, and do not use violet light for deactivation. This leaves Padron fully activated during imaging, a convenient way to compare linear one-step imaging directly against quadratic two-step imaging in the same sample.

### Two-step fluorescence improves image sectioning

**Figure 2:**
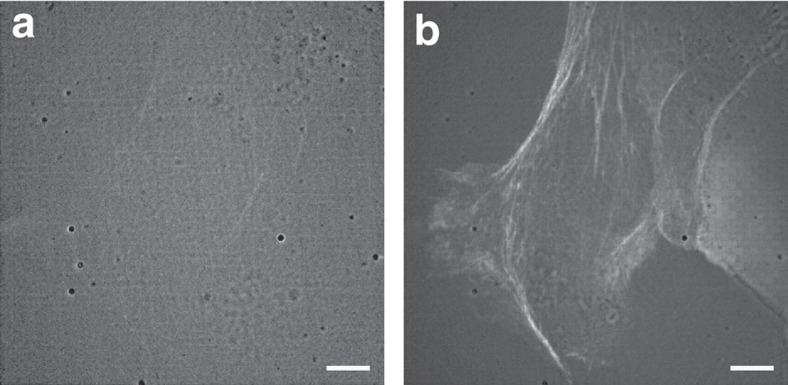
One-step versus two-step imaging of an artificial sample with substantial out-of-focus fluorophores. Fixed U2OS cells expressing F-tractin-Padron immersed in Padron solution, imaged with (**a**) one-step and (**b**) two-step fluorescence (see also Supplementary Fig. 2). Scale bars, 5 μm.

We expect quadratic non-linearity to improve image sectioning, so we compare one-step and two-step imaging of an artificial sample with and without out-of-focus fluorophores. Initially the sample is a single layer of fixed U2OS cells expressing an F-tractin-Padron fusion protein labelling the actin cytoskeleton^18^,^19^. As expected, one-step fluorescence produces high-quality images of this thin, sparsely fluorescent sample (Supplementary Fig. 2(e)). Next, we add an optically thick layer of Padron protein above the cells. As shown in Fig. 2a, one-step imaging becomes almost useless in this thick, densely fluorescent sample. However, two-step imaging recovers a high-quality image despite very unfavourable conditions (Fig. 2b).

Digital processing can remove smooth background signals due to out-of-focus fluorophores, but cannot remove Poisson noise due to this background. When out-of-focus noise swamps the in-focus signal, as it does here during one-step imaging, information is lost which digital background subtraction cannot recover (Supplementary Fig. 2). Two-step fluorescence creates far less out-of-focus excitation (Supplementary Fig. 3), which avoids the noise associated with high background, preventing information loss. This sample is an extreme case, but similar conditions might be found when imaging thick, densely tagged tissues like muscle or brain.

### Two-step fluorescence improves two-photon image sectioning

**Figure 3:**
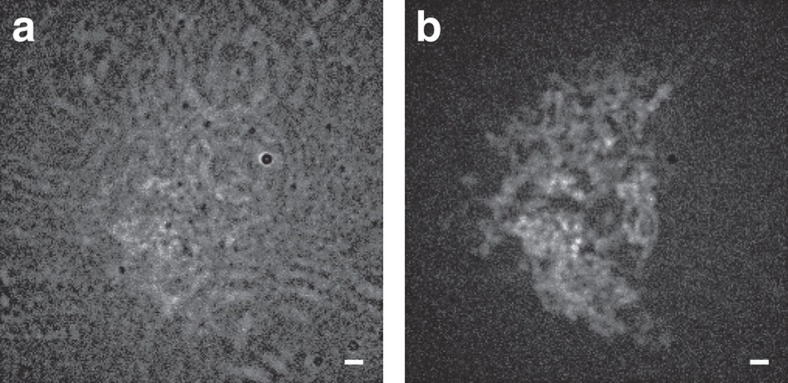
One-step, two-photon versus two-step, two-photon imaging of an artificial sample with substantial of out-of-focus fluorophores. Fixed U2OS cells with Padron-labelled mitochondria immersed in Padron solution, imaged with (**a**) one-step, two-photon and (**b**) two-step, two-photon fluorescence (see also Supplementary Fig. 5). Scale bars, 1 μm.

We expect quartic non-linearity to give even better sectioning than quadratic non-linearity, so we investigate the combination of two-step with two-photon fluorescence. We found that short pulses of infrared light (950 nm central wavelength, 140 fs pulse duration and 12.5 ns repetition time) efficiently activate and excite Padron (Supplementary Fig. 4(c)), so we compare one-step two-photon versus two-step two-photon imaging. We use a similar artificial sample to that shown in Fig. 2, and a similar imaging procedure except we substitute focused, pulsed infrared light for focused blue light. The initial sample is a single layer of fixed U2OS cells expressing Padron fused to the mitochondrial targeting sequence of the precursor of subunit VIII of human cytochrome *c* oxidase. As expected, one-step two-photon imaging gives a high-quality image in this case (Supplementary Fig. 5(e)). Next, we add a drop of Padron protein above the cells. As shown in Fig. 3a, one-step two-photon imaging fails to capture a useful image in this challenging sample, while two-step two-photon imaging performs well (Fig. 3b), presumably because two-step two-photon fluorescence further reduces out-of-focus excitation (Supplementary Fig. 6), reducing noise enough to allow imaging.

We were initially surprised by this failure of one-step two-photon imaging, since two-step one-photon imaging worked well in similar conditions, and both processes give quadratic non-linearity. However, the cross-section for one-step two-photon fluorescence is very low compared with two-step one-photon fluorescence. For Fig. 2b, we used 1 mW of 488 nm light with 20 ms exposure time, whereas for Fig. 3a, we used 1 W (average power) of pulsed 950 nm light with 50 ms exposure time and roughly one-fourth the illuminated area – our average power, peak power and exposure time increase by roughly 10^3^, 10^8^ and 2.5-fold, respectively. Despite this massive increase in light dose and light intensity, signal levels are higher in Fig. 2b than Fig. 3a, possibly explaining the difference in image quality. Further increasing the two-photon power to improve signal levels is impractical; 1 W illuminating such a small area is already quite high, and photodamage increases faster than fluorescence as two-photon power increases^20^.

### Two-step fluorescence improves image resolution

**Figure 4:**
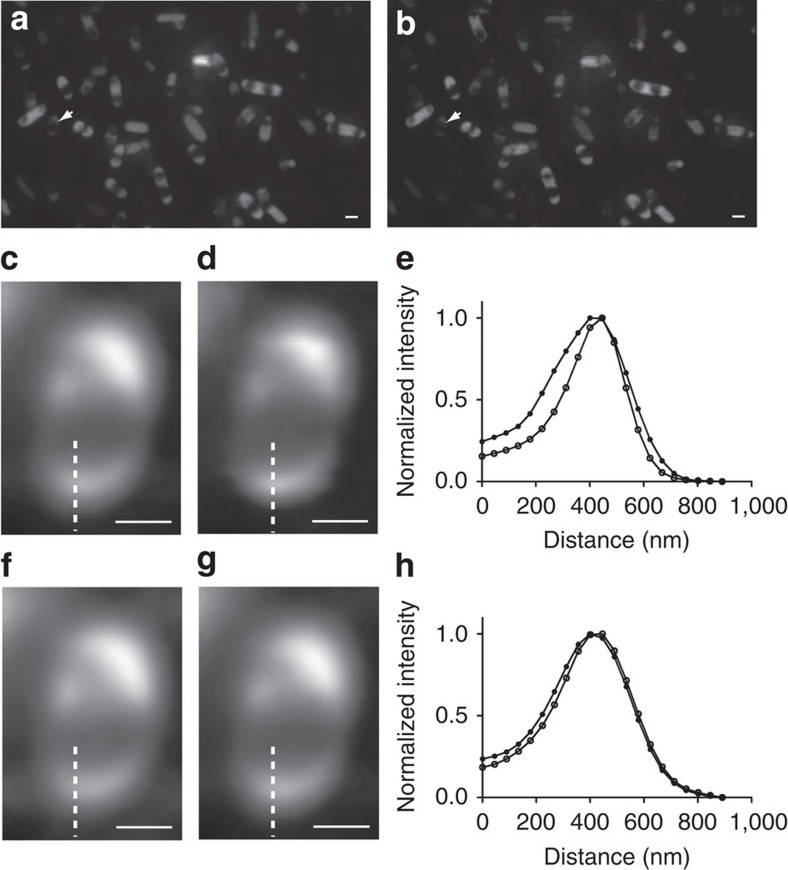
Two-step fluorescence improves image resolution by narrowing the excitation point-spread function. One-photon excitation images of BL21(λ)DE3 *Escherichia coli* bacteria expressing Padron using (**a**) one-step or (**b**) two-step fluorescence imaging. White arrows indicate the region expanded in (**c**,**d**). Intensity profiles measured along the white lines in (**c**,**d**) show that two-step fluorescence imaging ((**e**) open circles) narrows the apparent width of fine structures in excitation images when compared with one-step fluorescence imaging ((**e**) closed circles), consistent with our expectation that two-step fluorescence shrinks the excitation point-spread function. See Supplementary Fig. 7 for additional examples. The corresponding emission images (**f**,**g**) respectively, show almost no change in apparent width between one-step ((**h**) closed circles) and two-step ((**h**) open circles) imaging, consistent with our expectation that two-step fluorescence does not affect the emission point-spread function. Scale bars are 1 μm for (**a**,**b**) and 0.5 μm for (**c**,**d**,**f**,**g**).

We expect quadratic non-linearity to slightly shrink the excitation volume, improving the lateral resolution of an ‘excitation image’ (total intensity emitted from a single illumination spot versus illumination spot position). One-step (Fig. 4a) versus two-step (Fig. 4b) one-photon imaging of *Escherichia coli* expressing Padron qualitatively confirms this prediction. As expected, two-step imaging (Fig. 4d) produces a sharper excitation image than one-step imaging (Fig. 4c), narrowing the apparent width of fine structures (Fig. 4e, Supplementary Fig. 7 and Supplementary Software 1). Since the shape of the bacteria is three-dimensional, this effect could be due to either improved sectioning or improved lateral resolution. However, the corresponding emission images (total intensity collected at each camera pixel, Fig. 4f,g) show no apparent difference in width (Fig. 4h), implying that sectioning alone does not lead to narrowing. Stereotyped structures like microtubules or beads would be better for quantifying resolution, but these measurements are tricky because Padron’s two-step behaviour is sample-dependent.

Ideal inactive two-step fluorophores would not fluoresce, but inactive Padron fluoresces slightly. Inactive-state fluorescence is linear, which degrades quadratic resolution improvement (Supplementary Fig. 8(a) and Supplementary Software 2). Padron’s contrast (active divided by inactive fluorescence) can be ∼200 in bacteria^16^, high enough for nearly ideal resolution improvement. However, we find the contrast of Padron is strongly sample-dependent, ranging from 150 at high concentration to as low as 5 at low concentration (Supplementary Fig. 9). Unfortunately we found the contrast ratio of Padron-labelled beads or actin is too low to demonstrate resolution improvement, consistent with low local concentration of Padron.

## Discussion

Our data processing approach leaves room for improvement. In principle, a single camera exposure can collect a two-step fluorescence image; in practice, we collect two-step image data with many camera exposures at different illumination positions, because Padron requires violet deactivation light which causes undesired fluorescence. This raises the question, how best to combine these data into an image? Supplementary Figs 2 and 5 show individual data frames, sum projections and maximum-intensity projections (used in Figs 2 and 3), with and without digital background subtraction. These simple processing methods highlight the sectioning capability of two-step fluorescence imaging. Fig. 4, panels (a–d) show excitation images, meaning total fluorescence produced by a single illumination spot versus illumination spot position (Supplementary Software 1). We display the excitation image because the lateral resolution of a sum projection depends on the emission point-spread function, whereas two-step fluorescence improves the excitation point-spread function. More sophisticated data processing is possible but not required. For example, structured illumination algorithms^21^,^22^ (Supplementary Fig. 10 and Supplementary Methods) further improve image resolution by combining information from excitation and emission. We suspect the optimal way to fuse this data into a single image is iterative deconvolution^23^. Iterative deconvolution could also incorporate information from a time series at each scan position, but this would require precise characterization of the illumination and activation/deactivation kinetics. Finally, we expect ideal two-step fluorescence would be compatible with analogue processing^24^,^25^,^26^, although photoswitching kinetics might limit imaging speed.

Our demonstration of two-step fluorescent imaging was based on positive reversible photoswitching, meaning the excitation light also causes activation. Padron is fairly unusual in this regard; typical reversible probes are negative photoswitchers, meaning excitation light causes deactivation. Previous approaches exploit negative reversible photoswitching to improve sectioning, resolution or both^27^,^28^,^29^. These approaches are similar in spirit to two-step fluorescence, but with important differences, which highlight the advantages of positive photoswitching. Multiphoton deactivation and imaging (MPDI) and multiphoton activation and imaging (MPAI)^27^ both excite and switch Dronpa protein using two-photon illumination. MPDI activates with widefield 405 nm light, takes a two-photon image with focused, scanned 920 nm and then digitally subtracts background using a second image excited and deactivated with focused, scanned 920 nm. MPAI reduces background by deactivating with widefield 488, activating with focused, scanned 800 nm and exciting/deactivating with focused, scanned 920 nm. Cyclic sequential multiphoton (CSM) imaging^28^ excites and switches Dronpa protein using one-photon illumination, simultaneously scanning focused 405 and 488 nm beams through the sample. CSM digitally improves lateral resolution by modulating the 405 nm beam and subtracting low-activation signals from high-activation signals. Reversibly switchable photo-imprint microscopy (rsPIM)^29^ excites and switches either rsTagRFP or Alexa 647 using two visible colours. The imaging procedure is not yet disclosed, but presumably requires focusing both colours to a common spot and recording a time series, like CSM. rsPIM fits the multiexponentially decaying signal to a polynomial, and plots one of the polynomial coefficients versus position to produce an image.

Ideal two-step fluorescence is a promising alternative approach, with many appealing features. Two-step fluorescence improves lateral resolution, and works with both one-photon and two-photon illumination, unlike MPDI or MPAI. Two-step fluorescence prevents undesired excitation rather than digitally subtracting it, unlike MPDI, CSM and rsPIM; this is an important distinction, because digital subtraction removes background signal but not background noise (Supplementary Figs 2 and 5). The focused beam used for two-step fluorescence imaging is single-colour, unlike MPAI, CSM and rsPIM; this is also important, because sub-micron coalignment of focused beams with different colours is non-trivial due to chromatic aberration, especially while scanning large fields of view. Two-step fluorescence does not require recording a time series at each scan position, unlike CSM and rsPIM. Two-step fluorescence returns a signal which is proportional to the density of fluorophore, unlike rsPIM.

pcSOFI^30^,^31^ also exploits protein photoswitching to improve resolution and reject out-of-focus signal, but this is a subtle comparison, because SOFI measures a fundamentally different quantity than other microscopes. Microscope images are typically interpreted as a band-limited estimate of the density of fluorescently tagged molecules, but interpreting a SOFI image in this way would yield extremely misleading results, and this interpretation was never intended by SOFI’s inventors. There are many other important differences between SOFI and two-step fluorescence. SOFI operates at much lower activation levels, to reveal stochastic photoswitching events. SOFI uses widefield illumination, which prevents sectioned imaging via two-photon illumination, although this could conceivably be overcome via temporal focusing^32^. SOFI digitally subtracts out-of-focus signal rather than preventing its creation or detection, giving no rejection of background noise. Finally, SOFI inherently requires recording a time series followed by post processing, whereas a two-step image could conceivably be acquired in a single camera exposure (with sufficiently fast deactivation).

Two-step imaging has many advantages, but one glaring flaw: no true two-step fluorophore currently exists. Padron emulates ideal two-step behaviour at high concentrations and with violet illumination; this is enough to demonstrate the principle and promise of two-step fluorescent imaging, but is unsuitable for many applications. Imaging techniques which exploit unusual photophysics are rarely demonstrated via ideal fluorophores; the first stimulated emission depletion microscope used pyridine crystals^33^, reversible saturable optical fluorescence transitions microscopy used asFP595^34^, photoactivated localization microscopy used photoactivatable green fluorescent protein (PA-GFP) and dimeric Eos (dEOS)^6^,^8^, while stochastic optical reconstruction microscopy used Cy3–Cy5 dye pairs^7^. None of these fluorophores are popular today, but they spurred rapid development of their replacements by demonstrating the promise of their respective techniques. We hope Padron similarly demonstrates the promise of two-step imaging, and spurs similar development and replacement.

Mutagenesis of Padron is one promising path to a true two-step fluorophore, especially because to our knowledge, no attempt has been made to optimize Padron’s two-step behaviour. Recently, mutagenesis dramatically improved Padron’s reversible saturable optical fluorescence transitions microscopy performance^35^, suggesting similar improvements may be possible for two-step behaviour. A Padron mutant with higher contrast ratio at low concentrations would enable two-step imaging in a much wider variety of samples. Padron’s concentration-dependent contrast ratio suggests that oligomerization reduces inactive-state fluorescence, and we speculate that a Padron tandem dimer would have improved contrast. Reducing Padron’s activated lifetime (currently ∼2 h) is also highly desirable. The faster Padron turns itself off, the less violet illumination we need, enabling much gentler live imaging, and eliminating one mechanism of photobleaching cross-talk in multi-colour imaging. If deactivation were sufficiently fast, violet illumination could be eliminated, and simple raster scanning could replace interleaved scanning.

Positive photoswitchable proteins such as KFP1 (ref. 36) and rsCherry^37^,^38^ offer alternative starting points for mutagenesis. KFP1 activates and excites with 532 nm light, and spontaneously decays to the off state in seconds. rsCherry activates and excites with 550 nm light and deactivates with 450 nm light. However, rsCherry’s positive photoswitching only lasts a few cycles, KFP1 is a tetramer, and both proteins have low brightness and poor contrast ratio.

Screening existing fluorophores for positive photoswitching is also possible. In general, illumination causes some combination of activation, deactivation, excitation and bleaching. Mapping how these rates depend on wavelength, both one-photon and two-photon^39^, could identify other promising candidates for two-step fluorophores.

Mutagenesis is tedious, but the payoff could be high. A well-behaved two-step fluorophore would be preferable to one-step fluorophores for most cases. If two-step imaging proves compatible with three-photon imaging^40^, we expect a sextic non-linearity (proportional to intensity to the sixth power). Especially for challenging applications like live imaging in the mammal brain, the additional background suppression and scattering resistance from such a high non-linearity could prove invaluable.

## Methods

### Instrumentation and data collection

Data were collected on our multifocal structured illumination microscope (MSIM^14^), using a Nikon × 100 1.45NA oil objective. The instrument is equipped with a 50-mW 488-nm laser (Oxxius; LBX-488–50-CIR-PP) and a tunable multiphoton Ti:sapphire laser delivering 1 W at 950 nm (Coherent Inc.; Chameleon Vision II) which serve as one-photon or two-photon excitation sources. The excitation light is patterned by a microlens array (Thorlabs Inc., MLA150-5C) into a grid of point foci that can be scanned arbitrarily on the sample using galvanometric mirrors (Thorlabs Inc.; GVSM002) and imaged using an EM-CCD (Andor; iXon Ultra). An LED source (CoolLED; pE-2) delivers widefield 405 nm illumination to the microscope’s backport through a filter (Semrock; 390/482/587 nm BrightLine; FF01-390/482/587-25) and a 405 nm dichroic beamsplitter (Semrock; BrightLine single-edge laser-flat; Di02-R405) in the turret wheel diverts the light towards the sample. Micro-manager^41^ controls the microscope, including a shutter (Vincent Associates; LS 6Z2) in the two-photon beam. In experiments that use one-photon excitation, the camera signal triggers the acousto-optic tunable filter (AA Opto-Electronic Inc.; AOTFnC-400.650) which controls 488 nm dose.

An image of each focal plane was obtained by scanning the multi-foci grid over 200 positions with five images collected per scan position for two-step imaging. We divide the illumination dose across five camera exposures to allow detection of activation saturation (Supplementary Fig. 11), but since we did not observe saturation, we sum these five images to produce a single image per scan position. Coarse scans were interleaved to produce a finer scan to maximize the deactivation at each spot while minimizing the total 405 nm dose per slice. Unless noted, individual frames were recorded using a 20 ms exposure time and 1 mW of 488 nm laser excitation for one-photon experiments, or 1 W of 950 nm pulsed laser excitation for two-photon experiments (measured at the microscope’s side port). The illuminated area during imaging is ∼90 μm wide for 488 nm illumination, and ∼45 μm wide for 950 nm illumination. The microlens array decreases the fill-factor of the illumination by ∼27, but decreases throughput by ∼10, giving an overall increase in peak intensity of ∼2.7. For two-step imaging, after each galvo step the sample was deactivated for 50 ms using 30 μW of 405 nm widefield illumination measured at the objective (a lower bound on the power delivered to the sample). We estimate the area illuminated by the 405 nm light as ∼5,000 μm^2^, giving at least enough fluence to decrease the degree of activation of the sample by ∼50% after each galvo step^16^. Our simulations (Supplementary Fig. 8b) show this level of deactivation is sufficient to improve resolution. Acquiring each image slice (5 repetitions, 200 positions and 50 ms deactivation) took 62 s.

### Sample preparation and imaging

F-tractin Padron-labelled samples were prepared by transfecting U2OS cells (American Type Culture Collection) attached to no. 1.5 25-mm-diameter coverslips (Warner Instruments) with 1 μg of F-tractin-Padron, a plasmid derived from F-tractin-GFP^18^,^19^ and a gift from Drs Clare Waterman and Robert Fischer (National Heart, Lung and Blood Institute, National Institutes of Health). Samples were fixed with 4% paraformaldehyde in PBS buffer, quenched with 100 mM glycine in PBS, washed and dried. A thin layer of Cytoseal mounting medium (Electron Microscopy Sciences) was applied over the cells on the coverslips and allowed to dry. After cells were imaged, a drop of 157 μM fully activated Padron was placed on top of the cells. The Padron solution did not penetrate the cells due to the Cytoseal layer, but provided a large out-of-focus fluorescence background, effectively infinitely thick for imaging purposes (>1 mm). One-step imaging was performed first, followed by two-step imaging using 405 nm light to deactivate.

U2OS cells expressing Padron targeted to the mitochondria were prepared in the same manner using a plasmid obtained by exchanging PA-GFP with Padron. The plasmid expressed Padron fused to the mitochondrial targeting sequence of the precursor of subunit VIII of human cytochrome *C* oxidase. The cells were imaged in the same manner as above, except substituting focused 950 nm pulsed light for the focused 488 nm light.

Padron protein was purified as described previously^42^ using Padron-pQE31 plasmid,^16^ a gift from Dr Stefan Jakobs (Max Planck Institute). Bacterial samples for imaging were prepared by transforming the plasmid into BL21 strain and plating on ampicillin-agar plates containing isopropyl β-D-1-thiogalactopyranoside. The bacteria were scraped off, transferred onto a coverslip and imaged in one-step and two-step mode.

Sectioning curves were obtained using Padron or EGFP samples embedded in 20% PBS-acrylamide gel (Acros Organics; 330221000) in 120 μm deep wells (Grace BioLabs, 654004). A pinhole was positioned at the microscope’s excitation sideport to isolate a single illumination spot and avoid cross-talk. A set of five images with 20 msec exposure time were acquired at each axial position in steps of 0.2 μm for one-photon (488 nm excitation) or 0.05 μm for two-photon (950 nm pulsed excitation). For two-step imaging, the sample was deactivated at each axial position with 500 ms of 405 nm widefield illumination. We plot the derivative of the fluorescence intensity in a 128 by 128 pixel window surrounding the illumination spot as a function of axial position.

Power dependence curves were collected using Padron samples embedded in PBS-acrylamide gel in 120 μm deep wells with an exposure time of 20 msec. Microlenses were removed to give widefield excitation with either 488 (one-photon) or pulsed 950 nm (two-photon) light, and the power levels were measured with a labmax top power meter (Coherent, Inc.;1104622) at the microscope sideport. The protein in the field of view was deactivated with 2,000 ms of 405 nm widefield illumination before the start of a timecourse acquisition. Data were analysed by plotting the mean fluorescence intensity versus time, of a 20 by 20 pixel region centred on the illumination beam.

### Data Processing

Figures 2 and 3 were constructed by subtracting background using Fiji’s^43^ ‘rolling ball’ algorithm with a ball radius of 5, and maximum-intensity projecting the 200 images (alternative data manipulations are shown in Supplementary Figs 2 and 5). Excitation images in Fig. 4 were constructed by displaying total intensity emitted from a single illumination spot versus illumination spot position, as follows: First locate the position of each excitation focus using freely available Python software^22^. Next, isolate each spot by multiplying the multi-spot image by a Gaussian mask with a sigma of 2 pixels. Then sum the intensity in each spot and record the intensity and the spot position. Finally, interpolate the slightly irregular intensity versus position data to produce upsampled images on a Cartesian grid. The Python script implementing this algorithm is included in the Supplementary Software 1 and 2.

## Additional information

**How to cite this article:** Ingaramo, M. *et al*. Two-photon-like microscopy with orders-of-magnitude lower illumination intensity via two-step fluorescence. *Nat. Commun.* 6:8184 doi: 10.1038/ncomms9184 (2015).

## Supplementary Material

Supplementary Information Supplementary Figures 1-11, Supplementary Methods and Supplementary References.

Supplementary Software 1 Python code to construct excitation images.

Supplementary Software 2 Python code to simulate dependence of resolution improvement on contrast ratio and deactivation.
