# Supplementary material for: Two-photon-like microscopy with orders-of-magnitude lower illumination intensity via two-step fluorescence
Source: Nat Commun. 2015 Sep 3;6:8184. doi: 10.1038/ncomms9184 (PMC4559865; doi:10.1038/ncomms9184)
Supplement: Supplementary Information — Supplementary Figures 1-11, Supplementary Methods and Supplementary References. [file ncomms9184-s1.pdf]

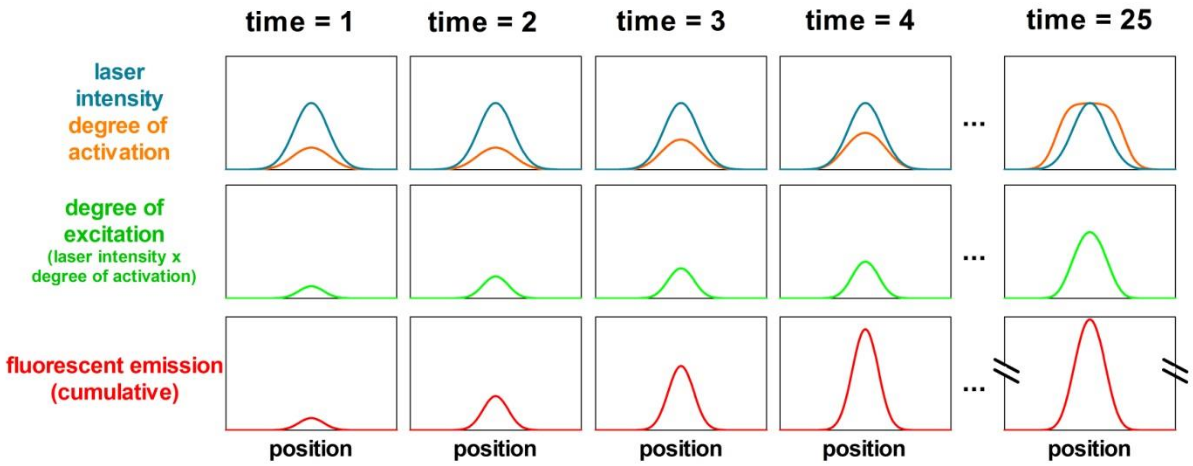


**Supplementary Figure 1. Cartoon illustration of the principle of two-step fluorescence microscopy.** These plots conceptually illustrate illumination, activation, excitation, and cumulative two-step signal vs. time during a single camera exposure. For our experiments, our activation/excitation illumination is point-focused, and constant in time during a single exposure. Times 1-4 illustrate a typical exposure, and time 25 illustrates the effects of activation saturation (if we were to dramatically increase our exposure time)

(First row, blue) For an ideal two-step fluorophore, the probability per unit time of activating an inactive fluorophore is proportional to the illumination intensity vs. position. The probability per unit time of exciting an active fluorophore is also proportional to the illumination intensity vs. position.

(First row, orange) In the limit where deactivation is negligible during activation, the inactive fraction of fluorophore decreases exponentially over the course of illumination. For small activation fractions, (times 1-4), the degree of activation vs. position is approximately proportional to the laser intensity vs postion, and also proportional to the illumination duration. Eventually (time 25), activation saturates, and these proportionalities become inaccurate.

(Second row, green) Since excitation and fluorescence emission reach equal-and-opposite equilibrium extremely rapidly, both the degree of excitation, the rate of excitation, and the rate of emission are proportional to the illumination intensity vs. position, for illumination intensities which do not saturate excitation. For an ideal two-step fluorophore, the inactive state does not fluoresce, so the degree of excitation and the amount of emission are also proportional to the degree of activation vs. position. Note that this implies the degree of excitation is proportional to the square of the illumination intensity.

(Third row, red) The cumulative fluorescent emission is simply the sum over time of the degree of excitation. If neither activation nor excitation saturate, the shape of the degree of excitation vs. position is roughly constant in time, and the cumulative fluorescent emission is proportional to the square of the illumination intensity.

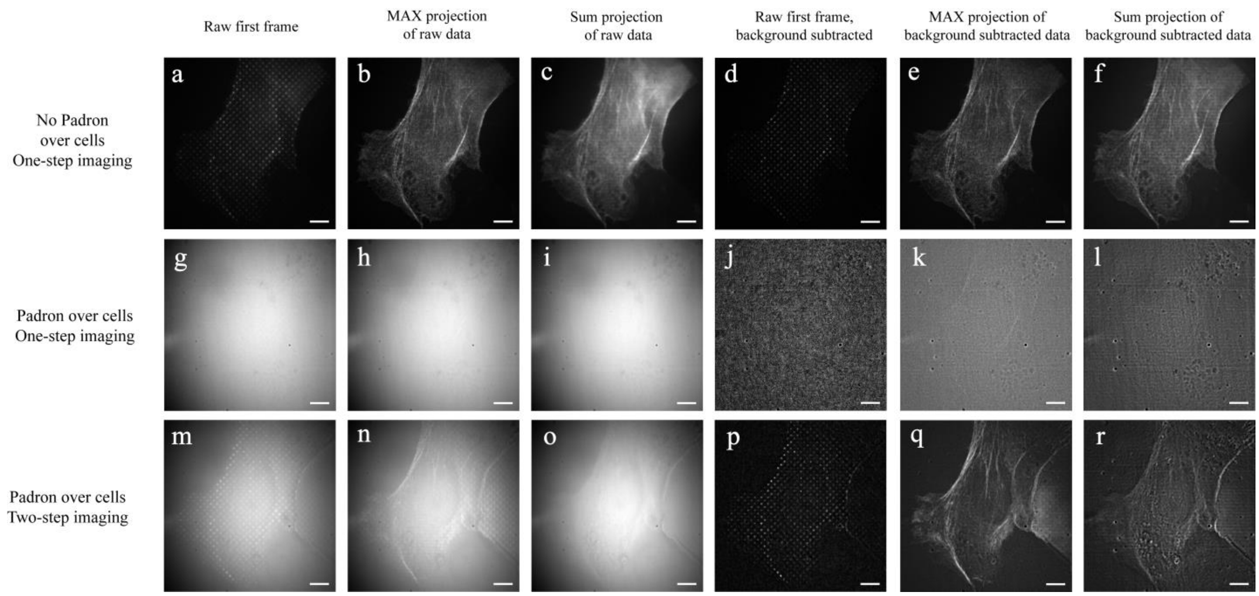


**Supplementary Figure 2. Comparison of simple visualizations for one-step and two-step fluorescence image data shown in Figure 2.** U2OS cells with Padron-labeled actin were fixed and sealed with a thin layer of Cytoseal mounting media, and imaged using one-step fluorescence (a-f). Next we add an optically thick layer of Padron above the cells, and image using one-step (g-l) and two-step fluorescence (m-r).

Each row shows different processing applied to the same data. The first row (a-f) shows the "ground truth", conventional one-step images before the extra Padron is added. The second row (g-l) shows how additional out-of-focus fluorophores disrupt one-step imaging. The third row (m-r) shows how two-step fluorescence recovers useful images despite the noisy background due to additional fluorophores.

Each column shows the same processing applied to different data. The first column (a, g, m) shows unprocessed frames obtained for a single position of the illumination spots. The second column (b, h, n) shows maximum intensity projections of 200 different positions of the illumination spots. The third column (c, i, o) shows summed intensity projections of 200 different positions of the illumination spots. The fourth, fifth, and sixth columns (d, j, p, and e, k, q, and f, l, r) show the same data as the first, second and third columns, respectively, except using simple digital background subtraction, Fiji's [1] "rolling ball" algorithm with a ball radius of 5. Panels (k) and (q) are shown in Figure 2 as panels (a) and (b), respectively. Scale bars: 5 µm.

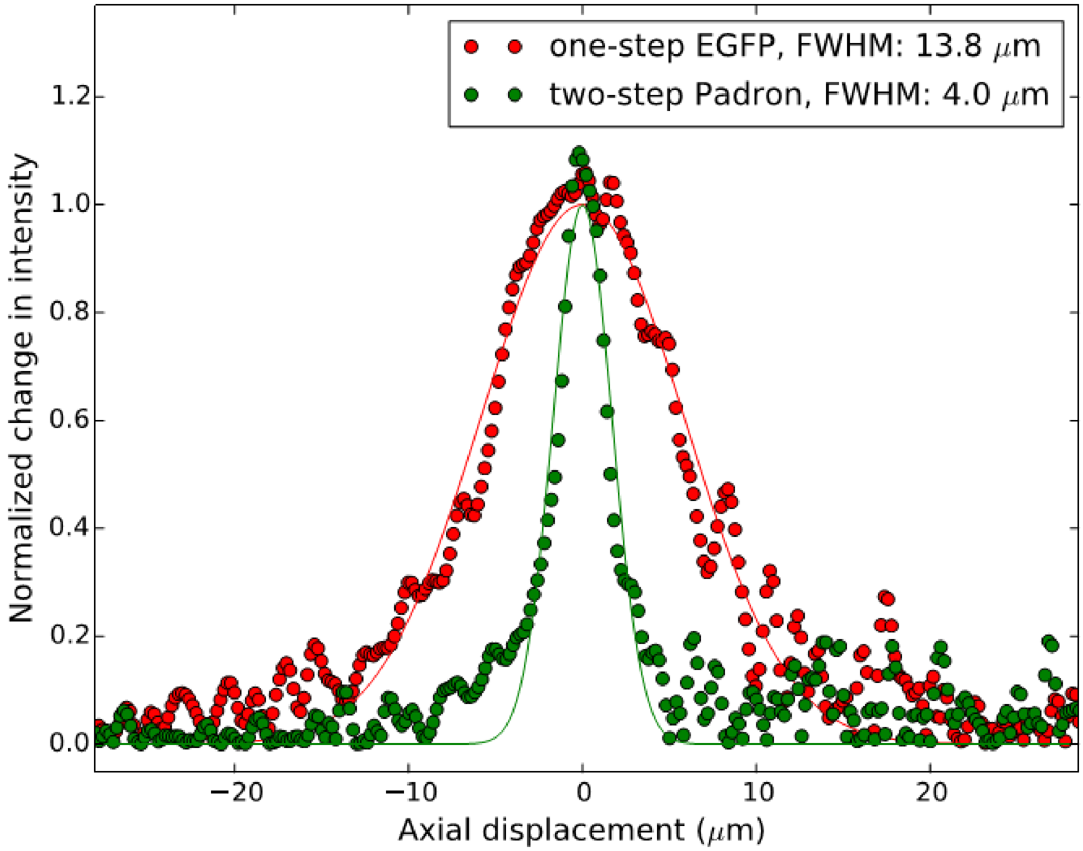


**Supplementary Figure 3. Two-step fluorescence imaging improves sectioning. 120 μm-deep wells were filled with either EGFP or Padron embedded in acrylamide gel.** We record mean signal intensity vs. axial position in a 128x128-pixel region of our camera centered on the illumination, and plot the derivative of this signal. We use a pinhole in the excitation path to isolate a single illumination spot, which eliminates crosstalk between spots. The zero position denotes the coverslip-sample interface. Two-step sectioning curves using Padron (green circles) show improved sectioning compared to one-step sectioning curves using EGFP (red circles). Solid lines show Gaussian fits, to approximately quantify the full-width at half-maximum (FWHM) of the sectioning curves. Note that the one-step curve only displays sectioning because of the finite pixel region analyzed; analyzing a larger region gives even worse sectioning for one-step, but also increases noise levels.

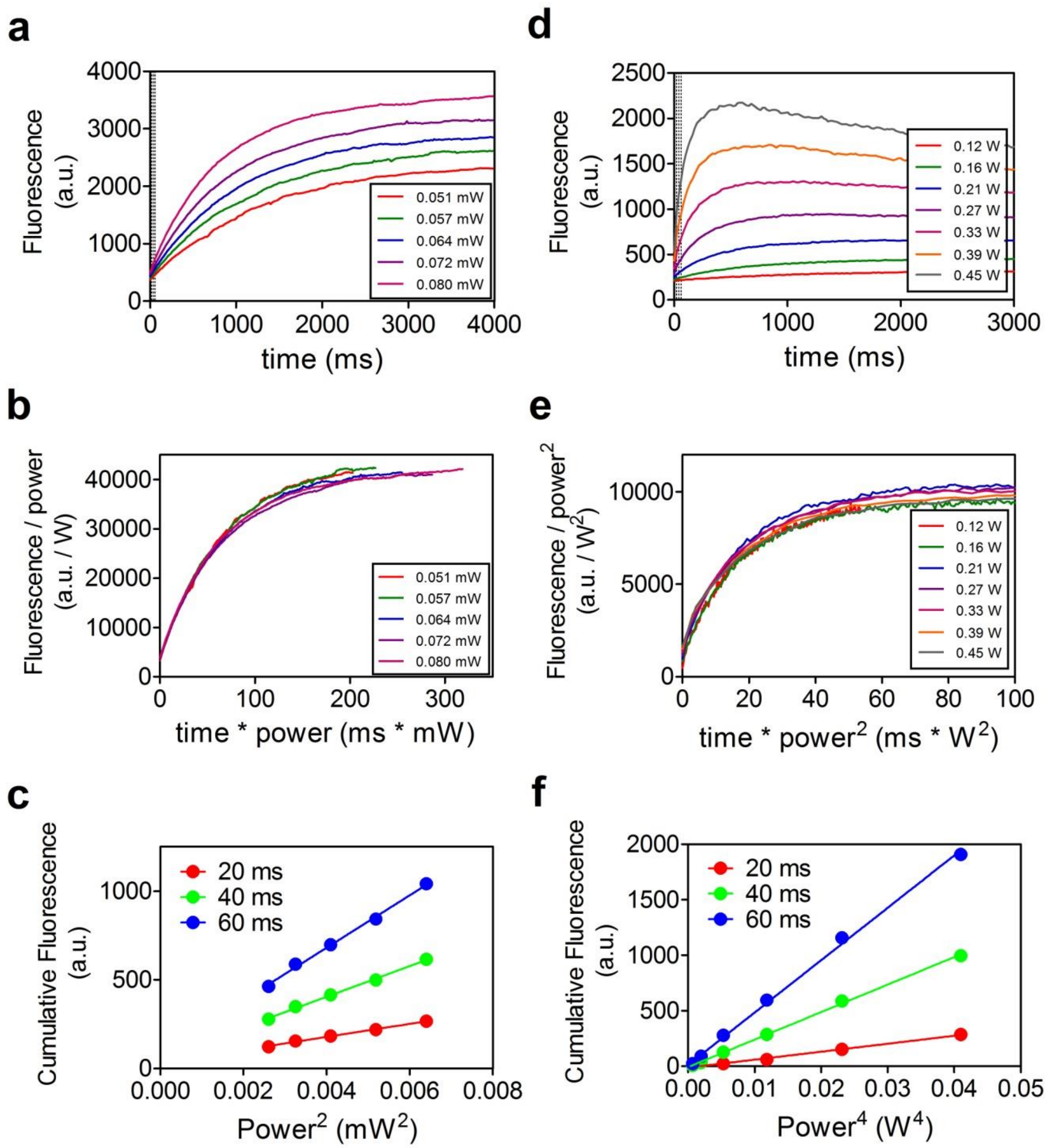


**Supplementary Figure 4. Both activation and excitation of Padron are proportional to one-photon power, and proportional to two-photon power-squared.** We illuminate 60 µM inactive Padron embedded in acrylamide gel with a collimated beam of either 488 (one-photon) or pulsed 950 (two-photon) nm light. We plot mean fluorescent signal vs. time in a 20x20 pixel region centered on the illumination, with 20 ms exposures. The 950 nm beam diameter is 3.6x smaller than the 488 nm beam. We expect one-photon activation and excitation (a) to both be proportional to 488 nm power, so we also plot the same data with fluorescent signal and time

scaled by 488 nm power levels (b); the curves collapse onto one another, as expected. (c) An alternative, simplified visualization of a small subset of this data demonstrates explicitly that total fluorescent signal is proportional to the square of the 488 nm power, for total exposure times of 20, 40, and 60 ms. We similarly expect two-photon activation and excitation (d) to both be proportional to the square of 950 nm power, so we also plot the same data with fluorescent signal and time scaled by the square of 950 nm power levels (e). Again, the curves collapse onto one another as expected. (f) An alternative, simplified visualization of a small subset of this data demonstrates explicitly that total fluorescent signal is proportional to the fourth power of the 950 nm power, for total exposure times of 20, 40, and 60 ms.

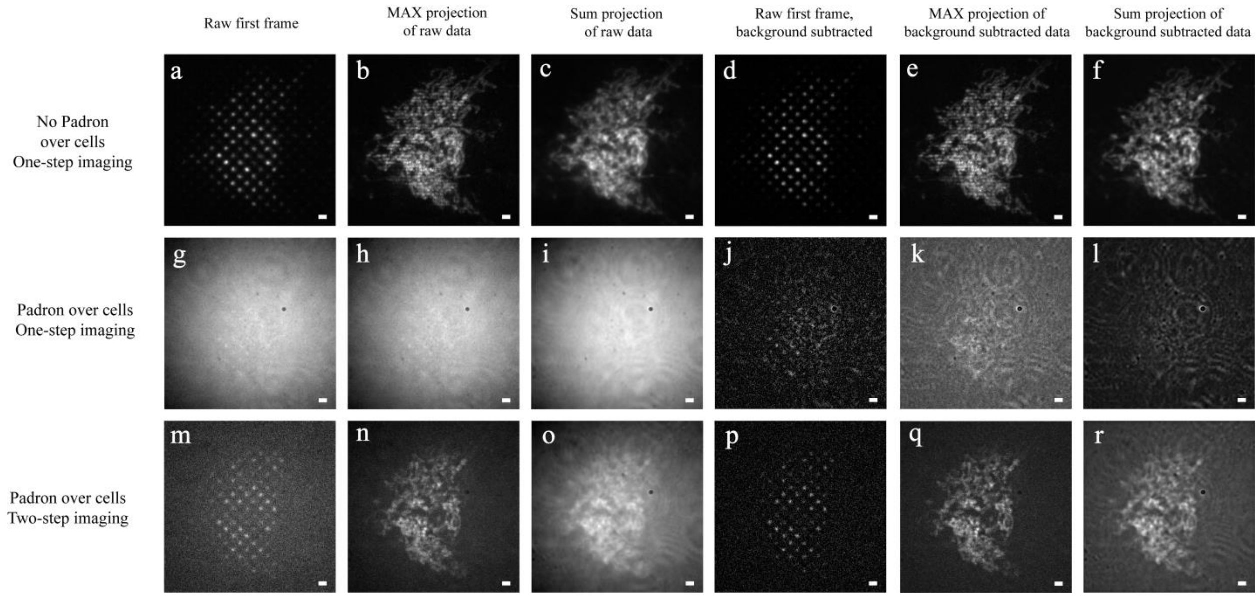


**Supplementary Figure 5. Comparison of simple visualizations for one-step, two-photon and two-step, two-photon fluorescence image data shown in Figure 3.** Similar to Figure S1, except using a different sample, and using 950 nm pulsed illumination instead of 488 nm illumination. U2OS cells with Padron-labeled mitochondria were fixed and sealed with a thin layer of Cytoseal mounting media, and imaged using one-step, two-photon fluorescence (a-f). Next we add an optically thick layer of Padron above the cells, and image using one-step two-photon (g-l) and two-step, two-photon fluorescence (m-r).

Each row shows different processing applied to the same data. The first row (a-f) shows the "ground truth", conventional one-step, two-photon images before the extra Padron is added. The second row (g-l) shows how additional out-of-focus fluorophores disrupt one-step, two-photon imaging. The third row (m-r) shows how two-step, two-photon fluorescence recovers useful images despite the noisy background due to additional fluorophores.

Each column shows the same processing applied to different data. The first column (a, g, m) shows unprocessed frames obtained for a single position of the illumination spots. The second

column (b, h, n) shows maximum intensity projections of 200 different positions of the illumination spots. The third column (c, i, o) shows summed intensity projections of 200 different positions of the illumination spots. The fourth, fifth, and sixth columns (d, j, p, and e, k, q, and f, l, r) show the same data as the first, second and third columns, respectively, except using simple digital background subtraction, Fiji's [1] "rolling ball" algorithm with a ball radius of 5. Panels (k) and (q) are shown in Figure 3 as panels (a) and (b), respectively. As with one-photon excitation, two-step two-photon imaging provides an improvement over one-step two-photon imaging regardless of background subtraction or projection method. Scale bars: 1 µm.

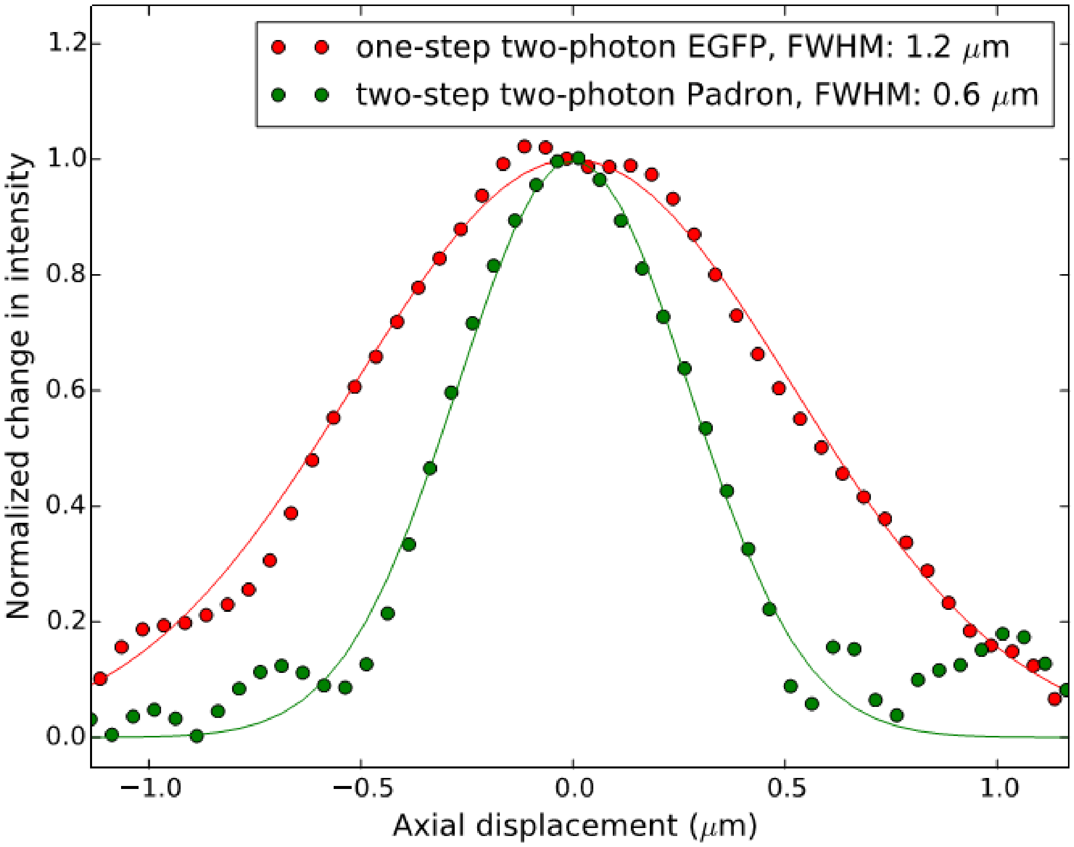


**Supplementary Figure 6. Two-step fluorescence further improves two-photon image sectioning. Similar** to Figure S2, except using 950 nm pulsed illumination instead of 488 nm illumination. 120 µm-deep wells were filled with either EGFP or Padron embedded in acrylamide gel. We record mean signal intensity vs. axial position in a 128x128-pixel region of our camera centered on the illumination, and plot the derivative of this signal. We use a pinhole in the excitation path to isolate a single illumination spot, which eliminates crosstalk between spots. The zero position denotes the coverslip-sample interface. Two-step two-photon sectioning

curves using Padron (green circles) show improved sectioning compared to one-step two-photon sectioning curves using EGFP (red circles). Solid lines show Gaussian fits, to approximately quantify the full-width at half-maximum (FWHM) of the sectioning curves.

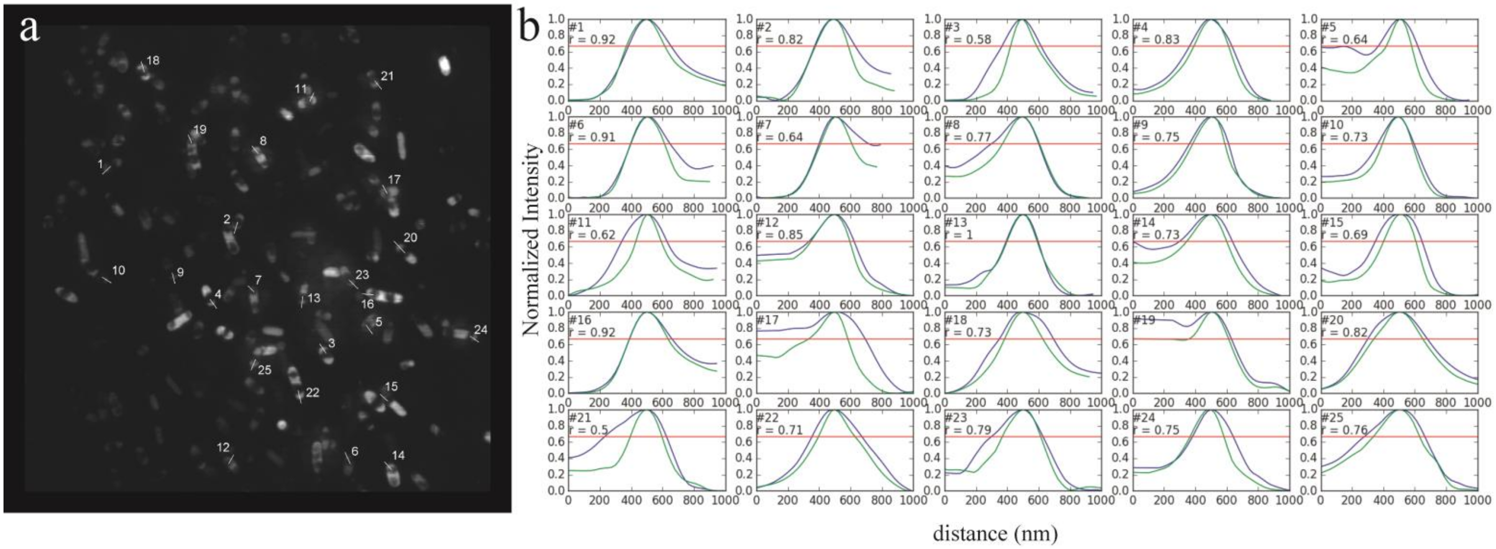


**Supplementary Figure 7. Two-step fluorescence improves image resolution.** Figure 4(e) shows a single example of how two-step fluorescence imaging narrows the apparent width of fine structures when compared to one-step fluorescence imaging; here we present 25 more such examples. (a) One-photon, two-step excitation image of BL21(λ)DE3 *E. coli* bacteria expressing Padron. Numbers indicate the line used for the intensity profiles plotted in (b), showing two-step fluorescence (green line) decreases the apparent width of fine structures when compared to one-step fluorescence (blue line). The red lines indicate the positions at which widths were estimated (full width third max). The ratio (r) of the two-step width over the one-step width is denoted on each plot; we observe a decrease in width of 0.76 ± 0.12 (mean ± SD, n=23), consistent with our expectations based on simulation (Supplementary Figure 8) (We also searched for any features that *increased* in width due to two-step imaging, but could not find any). This establishes that resolution is improved, but does not truly quantify resolution improvement, since the size of the features examined are unknown and presumably heterogeneous. More rigorous measurements would require an improved two-step fluorophore that does not suffer from Padron’s sample-dependent contrast ratio.

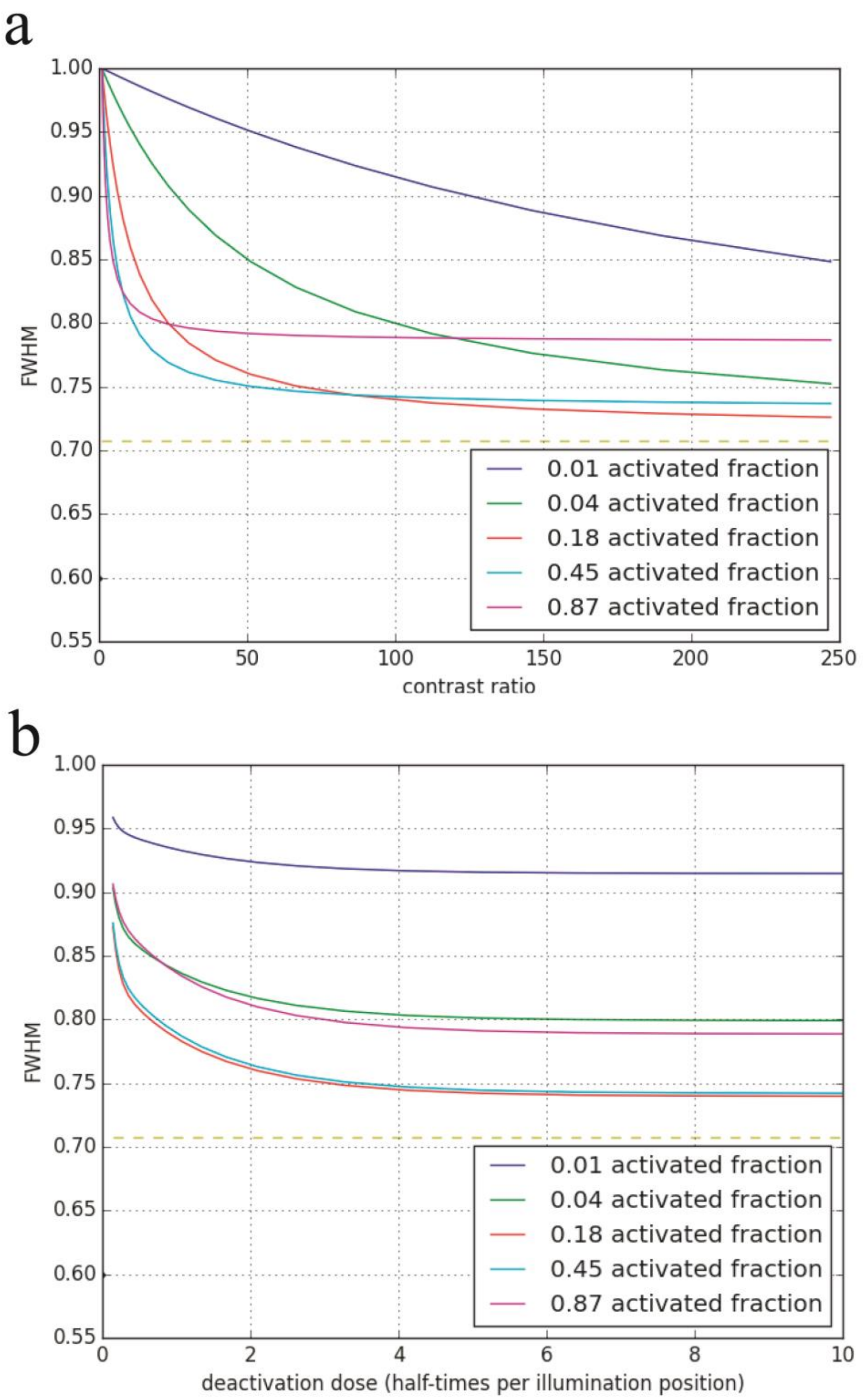


**Supplementary Figure 8. Simulated dependence of resolution improvement on contrast ratio and deactivation.** (a) An ideal two-step fluorophore has infinite contrast, meaning it does

not fluoresce in the inactive state. Real photoactivatable fluorophores fluoresce slightly in the inactive state; active-state fluorescence divided by inactive-state fluorescence is called the “contrast ratio”. Our simulations show resolution improvement from two-step fluorescence depends strongly on contrast ratio. With finite contrast, the measurement becomes dominated by the linear fluorescence of the off-state at sufficiently low dose. Higher dose increases the relative strength of the quadratic contribution, until dose is high enough to saturate activation and return to linear behavior. In order to maximize the contribution of the quadratic measurement, a high-contrast sample imaged with an appropriate light dose is critical for proof-of-principle resolution tests.

This simulation assumes complete deactivation after each illumination position, and we simulate several different levels of activation/excitation intensity. The simulated object is pointlike (one pixel wide), and we quantify resolution by plotting the full-width at half maximum (FWHM) of the simulated bead image vs. contrast ratio, normalized to the FWHM at contrast 0. Our illumination spot is approximated by a Gaussian. Each curve shown uses a different level of activation intensity, and is labeled by the peak activated fraction reached during imaging. The dashed line represents the $\sim\sqrt{2}$ resolution enhancement expected for a truly quadratic nonlinearity. Resolution improvement at low peak activation levels (lower illumination dose) requires very high contrast ratio. Resolution improvement at higher peak activation levels is more robust at low contrast, but worse at high contrast due to saturation.

(b) An ideal two-step fluorophore would deactivate rapidly and spontaneously, but Padron requires violet light for deactivation. This simulation assumes a contrast ratio of 100, and we simulate resolution improvement vs. deactivation dose, for several different levels of activation/excitation intensity. We describe the deactivation dose by how many deactivation switching half-times [2] the Padron experiences after each illumination position. The simulation is otherwise similar to the one described in (a). Our Python simulation code is provided as Supplementary Data File 2:

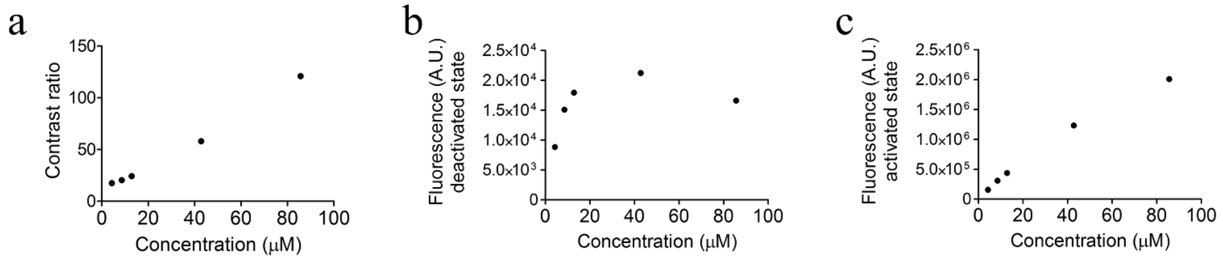


**Supplementary Figure 9. Concentration dependence of Padron contrast ratio.** We acquired fluorescence emission spectra in 10 µl cuvettes from 490 to 600 nm on a FluoroLog-3 spectrofluorometer (Horiba Scientific) using 475 nm excitation and 3 nm-wide excitation and emission bandwidth. We activate or deactivate Padron by illuminating the cuvette window for 20 seconds with either 130 mW of 405 nm laser or 65 mW of 488 nm laser. We calculate the contrast ratio (a) by dividing the fluorescence of the activated protein (c) emitted at 522 nm by the fluorescence of the deactivated protein (b) at the same wavelength. High contrast ratio (exceeding 100) only occurs at high protein concentrations.

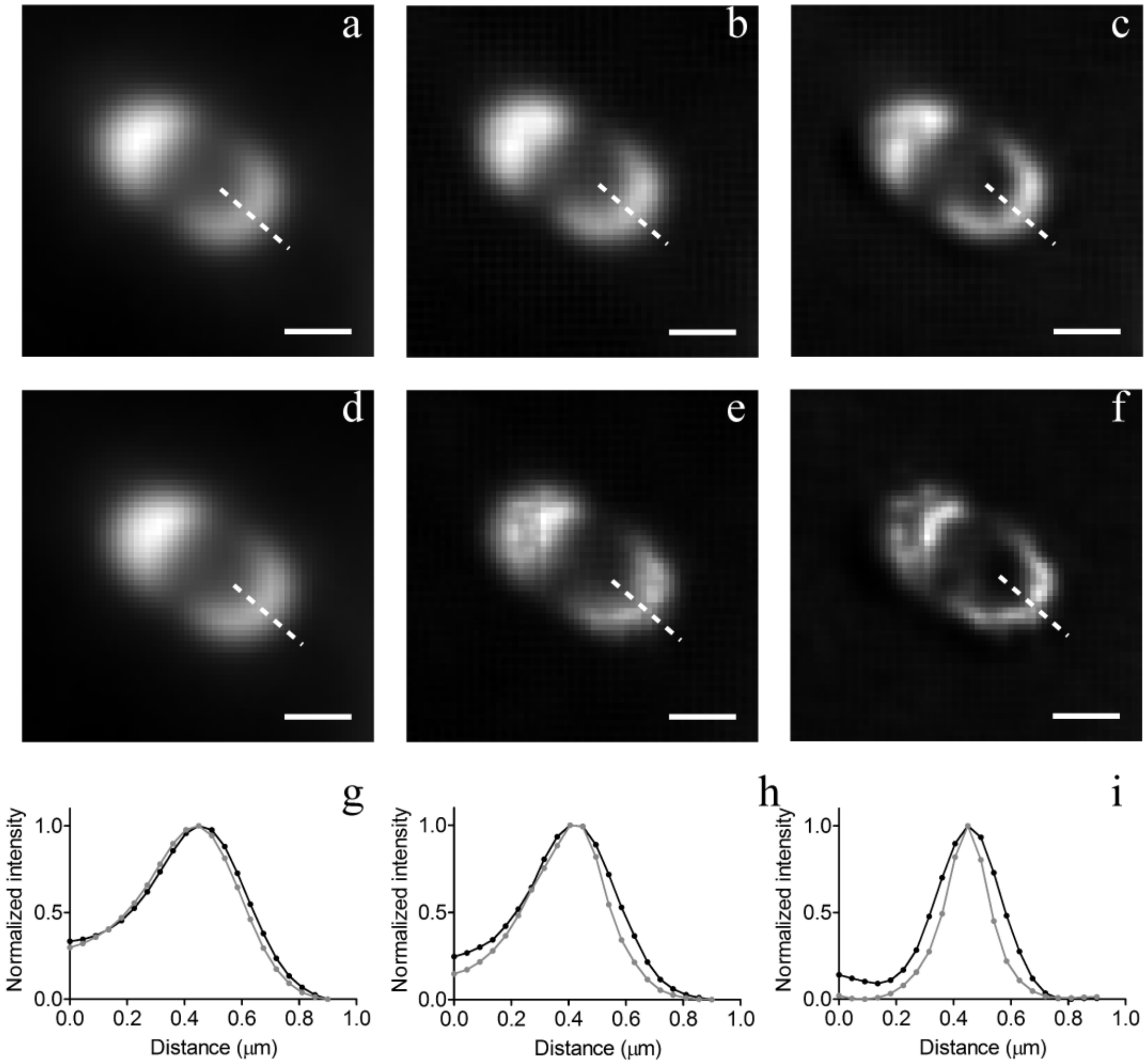


**Supplementary Figure 10. Two-step fluorescence improves structured illumination microscopy.** Data acquired in the same way as Figure 4, but processed with different methods. The top row shows one-step summed widefield (a), one-step MPSS (multifocal-excited, pinholed, scaled, and summed, (b)) and one-step MSIM (c) images, as described in [3]. The bottom row shows two-step images of the same bacteria, processed in the same way: two-step summed widefield (d), two-step MPSS (e) and two-step MSIM images (f). Intensity profiles (along the dotted lines in each panel) of the summed widefield (g), MPSS (h), and MSIM (i) images shows that two-step imaging (gray) narrows the apparent width of fine structures compared to one-step imaging (black) when combined with MPSS or MSIM. Scale bars: 0.5 μm.

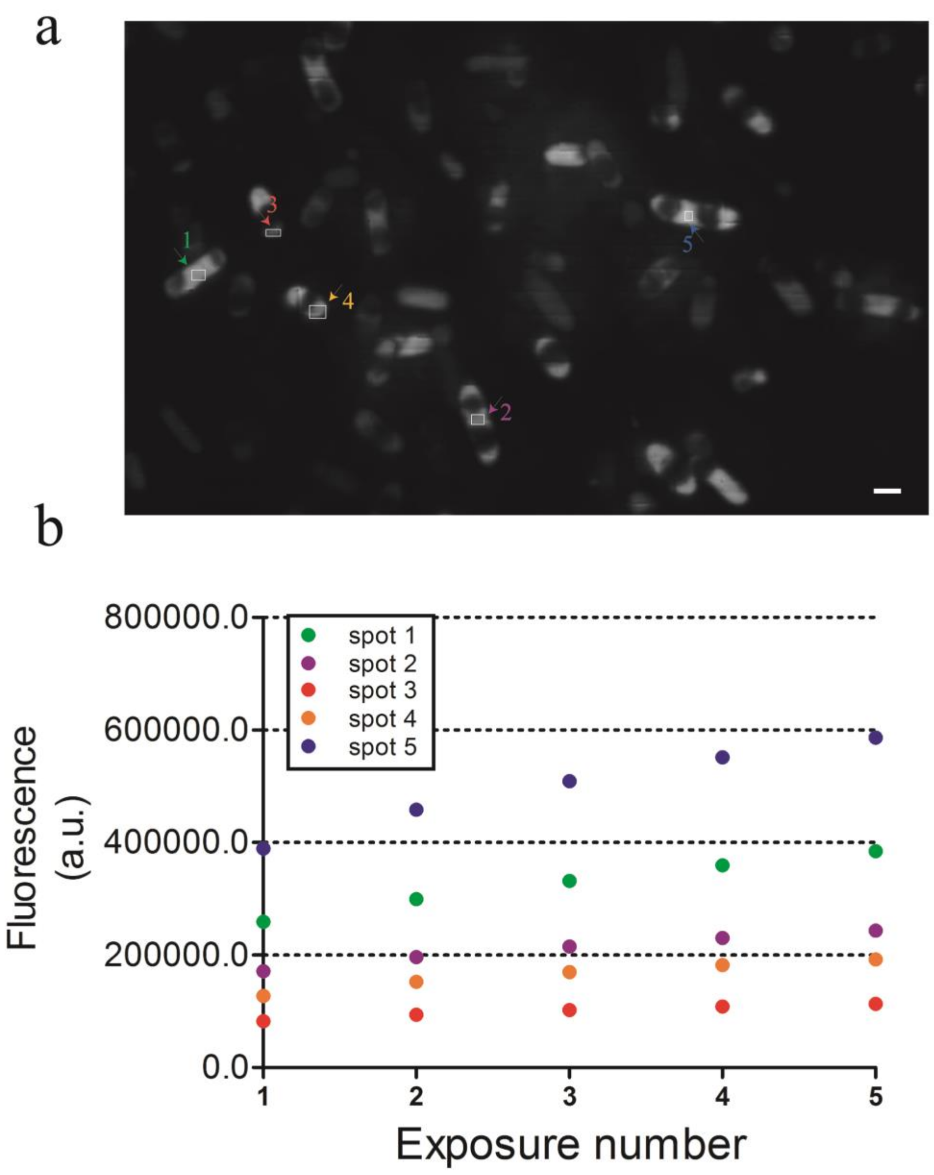


**Supplementary Figure 11. Fluorescence intensity vs. time during two-step imaging.** As described in the Methods section, we divide our two-step fluorescent signal across multiple camera exposures to rule out activation saturation. (a) For example, the two-step image from Figure 4 was collected using 5 consecutive 20 ms exposures at each galvo position, rather than a single 100 ms exposure. This allows us to inspect the fluorescent signal vs. time, as shown in (b) for each of the five labeled regions of (a), for signs of saturation. In each region, the fluorescent signal increases throughout the 100 ms exposure, and therefore activation has not saturated.

## SUPPLEMENTARY METHODS

### Multifocal structured illumination microscopy (MSIM) data processing

Data was processed with freely available software (https://code.google.com/p/msim/) as previously described [3], with the exception that a contraction factor of 0.33 was used instead of 0.5 to analyze the two step data, to account for the smaller excitation PSF [4].

REFERENCES


1 Schindelin, J. *et al.* Fiji: an open-source platform for biological-image analysis. *Nat Meth* **9**, 676-682 (2012).

2 Andresen, M. *et al.* Photoswitchable fluorescent proteins enable monochromatic multilabel imaging and dual color fluorescence nanoscopy. *Nat Biotechnol* **26**, 1035-1040 (2008).

3 York, A. G. *et al.* Resolution doubling in live, multicellular organisms via multifocal structured illumination microscopy. *Nat Methods* **9**, 749-754 (2012).

4 Ingaramo, M. *et al.* Two-photon excitation improves multifocal structured illumination microscopy in thick scattering tissue. *Proceedings of the National Academy of Sciences* **111**, 5254-5259 (2014).
