# Supplementary material for: Two-photon-like microscopy with orders-of-magnitude lower illumination intensity via two-step fluorescence
Source: Nat Commun. 2015 Sep 3;6:8184. doi: 10.1038/ncomms9184 (PMC4559865; doi:10.1038/ncomms9184)
Supplement: Supplementary Software 1 — Python code to construct excitation images. [file ncomms9184-s2.doc]

”””

Excitation imaging

Excitation images were constructed by displaying total intensity emitted from a single illumination spot vs. illumination spot position, using the following Python code:

”””

import os

import numpy as np

import matplotlib as plt

from scipy.interpolate import griddata

from simple_tif import array_to_tif, tif_to_array

from parameters import (direct_lattice_vectors,

corrected_shift_vector,

offset_vector)

from array_illumination import generate_lattice, get_shift

###########load data##############

def read_data(file_name, z, x, y):

return np.fromfile(file_name, dtype = np.uint16

).reshape((z,x,y)).astype(np.float32)

zpix=1000

filenames = ['linear_488ex_488em_Z_%05inm.raw'%i

for i in range(44192, 50025, 200)]

num_files = len(filenames)

num_repetitions = 5

for which_repetition in range(num_repetitions):

print "*"*20

print "Repetition:", which_repetition

print "*"*20

ex_stack = np.ones(((num_files), 512*8, 512*8), dtype=np.float64)

em_stack = np.ones(((num_files), 512*8, 512*8), dtype=np.float64)

for which_file, filename in enumerate(filenames):

print "Processing", filename

basename, ext = os.path.splitext(filename)

if ext == '.raw':

data = read_data(filename, zpix, 512, 512)

elif ext == '.tif':

data = tif_to_array(filename).astype(np.float32)

print data.shape

data = data[which_repetition::num_repetitions, :, :]

points = []

values = []

pinhole_radius = 20

mask_sigma = 2

grid_x, grid_y = np.mgrid[

0:data.shape[1]:8j*data.shape[1],

0:data.shape[2]:8j*data.shape[2]]

widefield_image = np.zeros(data.shape, dtype=np.float64)

print "zpix:",

for z in range(which_repetition, zpix, num_repetitions):

print z,

lattice = generate_lattice(

image_shape=data.shape[1:],

lattice_vectors=direct_lattice_vectors,

center_pix=offset_vector + get_shift(

corrected_shift_vector, z),

edge_buffer=pinhole_radius + 1)

normalization = 0

num_added = 0

widefield_contribution = []

new_values = []

slices = []

for x, y in lattice:

points.append((x, y))

x_slice = slice(round(x-pinhole_radius),

round(x+pinhole_radius+1))

y_slice = slice(round(y-pinhole_radius),

round(y+pinhole_radius+1))

slices.append((x_slice, y_slice))

x_coords = np.arange(x_slice.start - x, x_slice.stop - x, 1)

y_coords = np.arange(y_slice.start - y, y_slice.stop - y, 1)

x_mask = np.exp(-x_coords**2 / (2*mask_sigma**2)

).reshape(x_coords.size, 1)

y_mask = np.exp(-y_coords**2 / (2*mask_sigma**2)

).reshape(1, y_coords.size)

widefield_contribution.append(

data[z // num_repetitions, x_slice, y_slice] *

x_mask * y_mask)

new_values.append(widefield_contribution[-1].sum())

normalization += new_values[-1]

num_added += 1

normalization = normalization * 1.0 / num_added

for i, (x, y) in enumerate(lattice):

"""

Now that we have our normalization factor, normalize the

values we'll use for the excitation image, and add up the

contributions to the widefield image.

"""

new_values[i] = new_values[i] * 1.0 / normalization

widefield_image[

z // num_repetitions, slices[i][0], slices[i][1]] += (

widefield_contribution[i] * 1.0 / normalization)

values.extend(new_values)

array_to_tif(

widefield_image.reshape(

widefield_image.shape[0],

widefield_image.shape[1],

widefield_image.shape[2]).astype(np.float32),

basename +

'_repetition_%i_preprocessed_emission.tif'%(which_repetition))

widefield_image = widefield_image.sum(axis=0)

print "Interpolating excitation..."

interp_method = 'cubic'

interp_excitation_image = griddata(

np.asarray(points), np.asarray(values),

(grid_x, grid_y), method=interp_method)

print "Interpolating emission..."

widefield_points = np.array([(x, y)

for x in range(widefield_image.shape[0])

for y in range(widefield_image.shape[1])])

widefield_values = widefield_image.ravel()

interp_widefield_image = griddata(

widefield_points, widefield_values,

(grid_x, grid_y), method=interp_method)

print "Done interpolating."

ex_stack[which_file, :, :] = interp_excitation_image

em_stack[which_file, :, :] = interp_widefield_image

print "Saving..."

array_to_tif(ex_stack.astype(np.float32),

'excitation_repetition_%i.tif'%(which_repetition))

array_to_tif(em_stack.astype(np.float32),

'emission_repetition_%i.tif'%(which_repetition))

print "Done saving."
