# Supplementary material for: Two-photon-like microscopy with orders-of-magnitude lower illumination intensity via two-step fluorescence
Source: Nat Commun. 2015 Sep 3;6:8184. doi: 10.1038/ncomms9184 (PMC4559865; doi:10.1038/ncomms9184)
Supplement: Supplementary Software 2 — Python code to simulate dependence of resolution improvement on contrast ratio and deactivation. [file ncomms9184-s3.doc]

‘’’

Simulated dependence of resolution improvement on contrast ratio and deactivation

‘’’

import numpy as np

import matplotlib.pyplot as plt

from scipy.ndimage import gaussian_filter

from scipy.optimize.minpack import curve_fit

import simple_tif #available at https://code.google.com/p/msim/

def padron_bead_image(

activation_dose, #Watch out for saturation

contrast_ratio, #Higher is better

deactivation_dose, #Higher is better

num_steps, #Higher is more accurate but slower

save_output=True):

density_shape = np.array((1, 20, 20))

density = np.zeros(density_shape, dtype=np.float64)

bead_z = density_shape[0] // 2

bead_y = density_shape[1] // 2

bead_x = density_shape[2] // 2

density[bead_z, bead_y, bead_x] = 1

degree_of_activation = np.zeros_like(density)

degree_of_activation_max = 0

scan_step_size = np.array((1, 2, 2))

excitation_image = np.zeros(density_shape / scan_step_size,

dtype=np.float64)

def density_units_to_scan_units(z, y, x):

return np.array((z, y, x)) / scan_step_size

def scan_units_to_density_units(z, y, x):

return np.array((z, y, x)) * scan_step_size

coarse_step = 3

fine_step = 1

xy_scan_pattern = [] #scan units

for y_start in range(0, coarse_step, fine_step):

for x_start in range(0, coarse_step, fine_step):

for y in range(y_start,

density_shape[1] // scan_step_size [1],

coarse_step):

for x in range(x_start,

density_shape[2] // scan_step_size[2],

coarse_step):

xy_scan_pattern.append(np.array((x, y)))

def calculate_excitation(z, y, x, output): #Slow but easy to code

output.fill(0)

output[z, y, x] = activation_dose * 1.0 / num_steps

gaussian_filter(output, sigma=2, output=output) #Watch out near edges

return output

excitation = np.zeros_like(density)

which_file = 0

which_illumination = 0

for z_scan in range(excitation_image.shape[0]):

for x_scan, y_scan in xy_scan_pattern:

z, y, x = scan_units_to_density_units(z_scan, y_scan, x_scan)

"""

Determine excitation pattern

"""

calculate_excitation(z, y, x, output=excitation)

if save_output:

simple_tif.array_to_tif(

excitation.astype(np.float32),

'./illumination/excitation_%06i.tif'%which_illumination)

which_illumination += 1

for time in range(num_steps):

"""

Modify degree of activation

"""

unactivated = 1. - degree_of_activation

degree_of_activation += unactivated * excitation

current_max = degree_of_activation.max()

if current_max > 1:

print current_max

raise UserWarning("Use more steps")

degree_of_activation_max = max(

degree_of_activation_max,

degree_of_activation[bead_z, bead_y, bead_x])

"""

Accumulate signal into excitation image

"""

unactivated = 1 - degree_of_activation

glow = excitation * density * (

degree_of_activation + #Activated signal

unactivated * (1.0/contrast_ratio) #Unactivated signal

)

signal = glow.sum()

excitation_image[z_scan, y_scan, x_scan] += signal

if save_output:

simple_tif.array_to_tif(

excitation_image.astype(np.float32),

'./excitation/excitation_%06i.tif'%which_file)

simple_tif.array_to_tif(

degree_of_activation.astype(np.float32),

'./activation/activation_%06i.tif'%which_file)

which_file += 1

"""

De-activate

"""

for time in range(num_steps):

degree_of_activation -= (

degree_of_activation * deactivation_dose * 1.0 / num_steps)

if degree_of_activation.min() < 0:

raise UserWarning('increase num_steps!')

return (excitation_image, degree_of_activation_max)

def gauss_func(t, A, SD, mean):

return (A * np.exp(-0.5*((t-mean)/SD*(t-mean)/SD)))

def quantify_resolution(bead_image,

save_plots=False):

z_max, y_max, x_max = np.unravel_index(bead_image.argmax(),

bead_image.shape)

x_intensity = bead_image[0, :, x_max]

y_intensity = bead_image[0, y_max, :]

x_coords = np.arange(bead_image.shape[2])

y_coords = np.arange(bead_image.shape[1])

try:

popt_x, pcov = curve_fit(

gauss_func,

x_coords,

x_intensity,

p0=[x_intensity.max(), #A

0.8, #standard deviation

x_max]) #mean position

except RuntimeError:

popt_x=[0, 0, 0]

print 'failed fit'

try:

popt_y, pcov = curve_fit(

gauss_func,

y_coords,

y_intensity,

p0=[y_intensity.max(), #A

0.8, #standard deviation

y_max]) #mean position

except RuntimeError:

popt_y=[0, 0, 0]

print 'failed fit'

if save_plots:

plt.close('all')

fig = plt.figure()

plt.subplot(1, 2, 1)

plt.plot(x_coords, x_intensity, '.')

plt.plot(x_coords, gauss_func(x_coords, *popt_x), '-')

plt.subplot(1, 2, 2)

plt.plot(y_coords, y_intensity, '.')

plt.plot(y_coords, gauss_func(y_coords, *popt_y), '-')

fig.savefig('test_fitting.png')

return popt_x, popt_y

if __name__ == '__main__':

print "Calculating..."

a = quantify_resolution(

padron_bead_image(

activation_dose=5,

contrast_ratio=200,

deactivation_dose=1,

num_steps=100,

save_output=False

)[0],

save_plots=True)

print "Fit output:"

print a

print "Done."
